# Price of a Dog Bite: Multiorgan Failure Secondary to Capnocytophaga canimorsus Infection Requiring Veno-Venous Extracorporeal Membrane Oxygenation (VV-ECMO) Support

**DOI:** 10.7759/cureus.108881

**Published:** 2026-05-15

**Authors:** Juan D Salazar Borbón, Daniel Xu Carranza, Pablo A Alvarez Aguilar, Luis Quiros Figueroa

**Affiliations:** 1 Critical Care Medicine, Hospital San Juan de Dios, San José, CRI; 2 Critical Care Medicine, Hospital Mexico, San José, CRI; 3 Critical Care Medicine, Universidad de Costa Rica, San José, CRI; 4 Internal Medicine, Hospital Mexico, San José, CRI

**Keywords:** acute respiratory distress syndrome (ards), capnocytophaga canimorsus, clinical case report, multiorgan system failure, severe sepsis, venovenous extracorporeal membrane oxygenation

## Abstract

*Capnocytophaga canimorsus* is a zoonotic Gram-negative bacterium commonly present in the oral flora of dogs and cats. Although human infection is uncommon, it can lead to rapidly progressive and life-threatening disease, particularly in immunocompromised individuals such as those with asplenia. Severe acute respiratory distress syndrome (ARDS) requiring extracorporeal membrane oxygenation (ECMO) has been rarely reported.

We describe a 60-year-old woman with hypothyroidism and a remote history of splenectomy who presented with fever, malaise, and gastrointestinal symptoms one week after a dog bite. Blood cultures obtained early during hospitalization grew *C. canimorsus*. Her clinical course was complicated by rapidly progressive ARDS and multiorgan failure, requiring invasive mechanical ventilation. Despite optimized lung-protective ventilation, neuromuscular blockade, prone positioning, and broad-spectrum antimicrobial therapy, refractory hypoxemia persisted. Veno-venous (VV) ECMO was initiated as rescue therapy, resulting in progressive improvement in gas exchange and pulmonary mechanics. ECMO support was successfully discontinued after 11 days, followed by liberation from mechanical ventilation and full clinical recovery.

Severe *C. canimorsus* infection can result in fulminant ARDS and multiorgan dysfunction. Early recognition and timely escalation to advanced supportive therapies, including VV-ECMO, may be lifesaving in selected patients.

## Introduction

Zoonotic infections can be a significant clinical challenge due to their potential severity and the difficulty of diagnosis. *Capnocytophaga canimorsus* is a Gram-negative facultative anaerobic bacterium that colonizes the oral cavity of dogs and cats and can be transmitted to humans through bites. Although this species is generally considered to have low virulence in healthy individuals, previous studies have reported severe infections in patients with risk factors such as asplenia, alcoholism, cirrhosis, and immunosuppression, as well as in previously immunocompetent individuals [1-3].

*C. canimorsus* may be responsible for significant human infections following its transmission, mainly via dog bites. Transmission has also been described following scratches, saliva exposure, and even close contact with direct exposure of human mucosa to dog saliva, such as licking of the patient’s face in the absence of overt injury. Infection with *C. canimorsus* can be fatal, especially in the setting of sepsis in elderly or immunocompromised patients. Typical clinical manifestations of *C. canimorsus* infection include sepsis, disseminated intravascular coagulation, endocarditis, meningitis, and gangrenous necrosis of the digits [4].

Respiratory involvement is less commonly described; a few reports have documented *C. canimorsus* infection presenting with fever and pneumonia-like syndromes, predominantly in immunocompromised hosts [4]. Severe presentations may progress rapidly to septic shock and multiorgan failure (MOF), underscoring the importance of early recognition and prompt antimicrobial therapy.

Here, we describe an asplenic patient who developed severe acute respiratory distress syndrome (ARDS) secondary to *C. canimorsus* bacteremia one week after a dog bite on the hand. The patient required support by veno-venous extracorporeal membrane oxygenation (VV-ECMO), a clinical scenario rarely described in the literature.

## Case presentation

**Figure 1:**
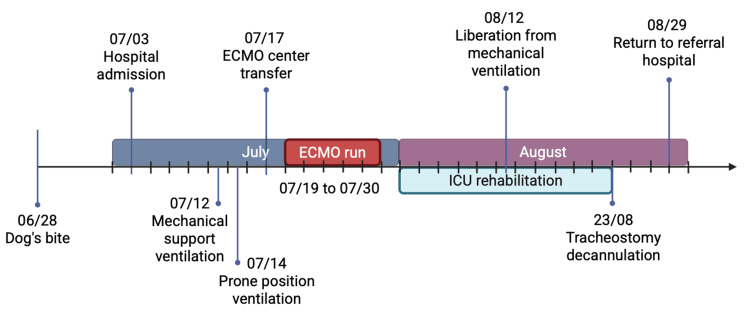
Timeline of clinical and organic support events. ICU: intensive care unit; ECMO: extracorporeal membrane oxygenation

A 60-year-old woman from Liberia, Costa Rica, presented in July 2025 with fever, malaise, and nausea one week after a dog bite (Figure 1). The patient had a history of hypothyroidism, sulfa allergy, and a previous abdominal surgery, which a review of her records indicated was a splenectomy more than 10 years previously. No relevant family history of infectious or chronic diseases was reported. The patient reported progressive constitutional symptoms several days after a dog bite, including fever, generalized malaise, and nausea.

Laboratory investigations at admission demonstrated normocytic-normochromic anemia, mild thrombocytopenia, leukocytosis with neutrophil predominance without bandemia, elevated blood urea nitrogen with a prerenal pattern in the absence of intrinsic renal injury, hypoalbuminemia, and elevated inflammatory markers, including C-reactive protein and procalcitonin (Table 1). Liver function tests and coagulation parameters remained within normal limits. Serologic testing for hepatitis B and C, syphilis, and HIV was negative.

**Table 1:** Serial laboratory findings and organ dysfunction progression during the first week of hospitalization. Arrows indicate decreased (↓) and increased (↑) values relative to the reference range. SOFA: Sequential Organ Failure Assessment

Parameter	Reference range	Admission	First week follow-up
Hemoglobin	12-16 g/dL	7.2 ↓	-
Platelet count	150,000-450,000/µL	134,000 ↓	-
White blood cells	4,000-10,000/µL	16,800 ↑	-
Neutrophils	1,500-7,500/µL	15,000 ↑	-
Blood urea nitrogen	7-20 mg/dL	50 ↑	-
Albumin	3.5-5.0 g/dL	1.7 ↓	-
C-reactive protein	<5 mg/L	240 ↑	-
Procalcitonin	<0.05 ng/mL	2.8 ↑	19 ↑
SOFA score	0-24	3	6

Serial laboratory assessments during the first week of hospitalization demonstrated progressive clinical deterioration, reflected by worsening inflammatory markers and an increase in the Sequential Organ Failure Assessment (SOFA) score from 3 to 6 (Table 1). Blood cultures were obtained on the day of admission. After three days of incubation, a Gram-negative bacillus was identified; however, it required referral to a tertiary care center for definitive identification. The diagnosis of *C. canimorsus* was therefore established five days after admission. At 14 days after admission, the patient had respiratory acidosis (pH 7.2, pCO_2_: 54 mmHg) and a PaO_2_/FiO_2_ of 93, consistent with severe ARDS. Antimicrobial susceptibility testing (AST) specific for *C. canimorsus* was not locally available, which may have contributed to delays in targeted therapy and reflects real-world limitations in resource-constrained settings.

**Figure 2:**
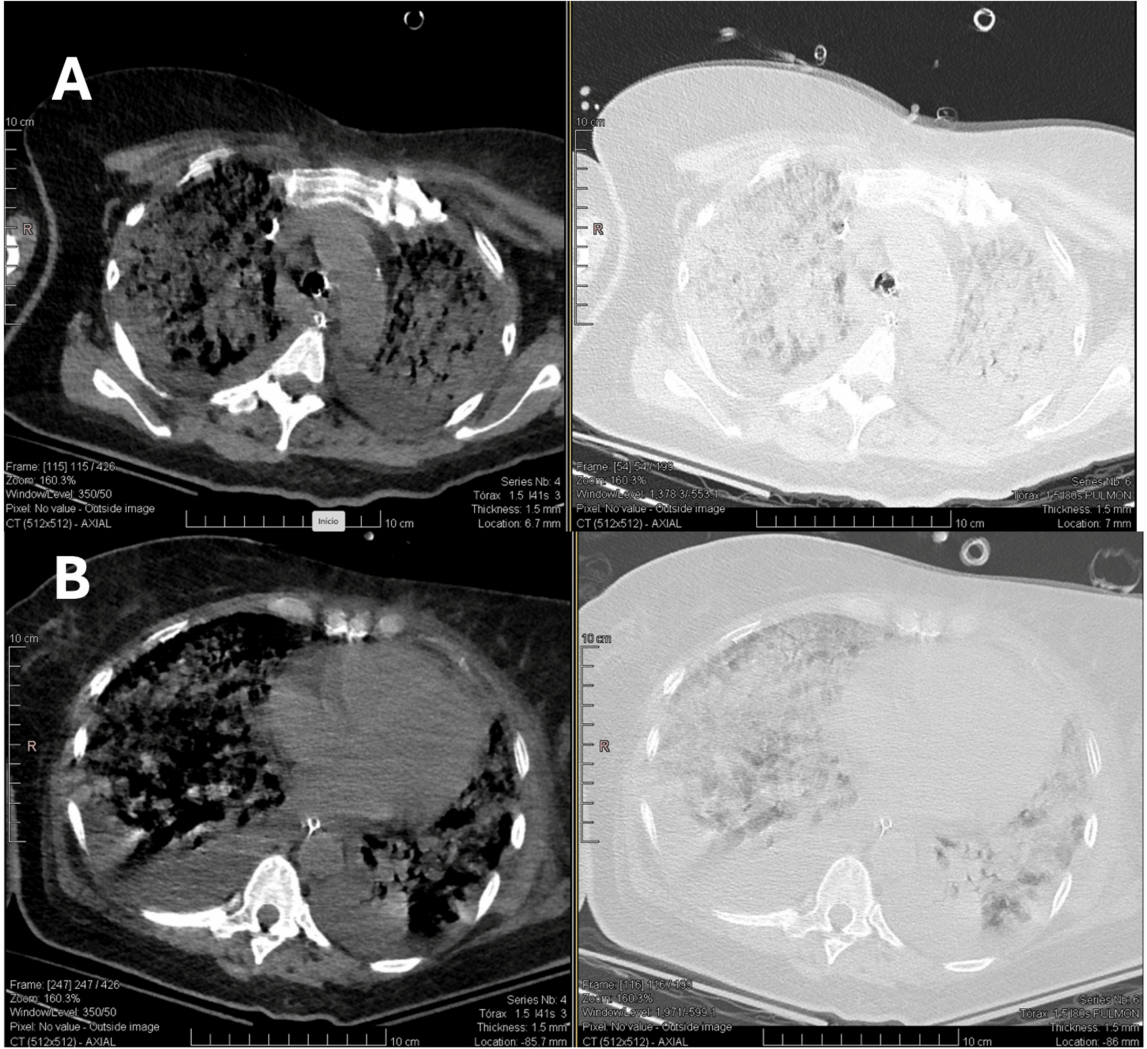
Chest computed tomography 1 day prior to the initiation of VV-ECMO therapy demonstrating diffuse bilateral infiltrates consistent with severe ARDS. (A) Axial image at the level of the aortic arch. (B) Axial image at the level of the atria. VV-ECMO: veno-venous extracorporeal membrane oxygenation; ARDS: acute respiratory distress syndrome

An initial chest ultrasound revealed minimal pleural effusion, and the abdominal findings were consistent with acalculous cholecystitis (data not shown). Chest computed tomography imaging (Figure 2) prior to VV-ECMO demonstrated diffuse bilateral infiltrates consistent with severe ARDS, but there was no evidence of pulmonary thromboembolism or enlargement of the cardiac chamber.

**Figure 3:**
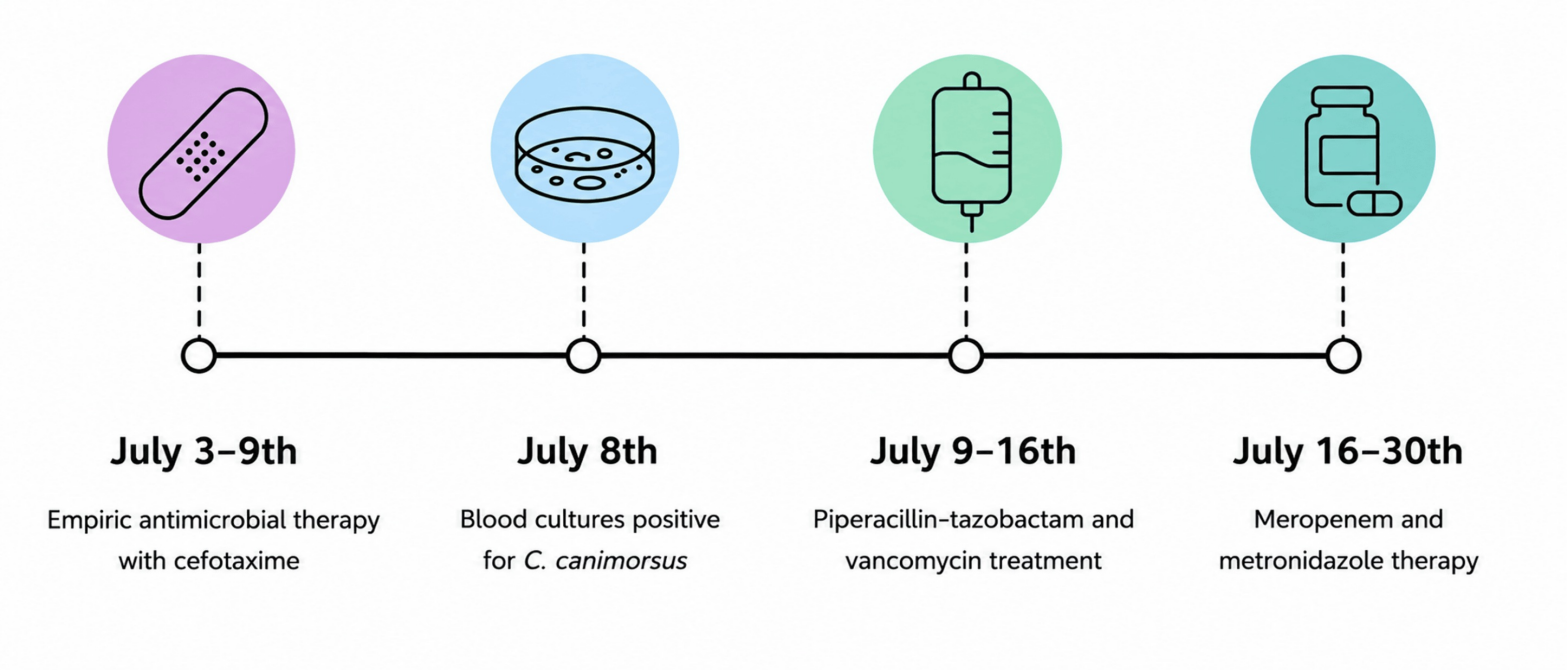
Timeline of microbiological findings and antibiotic therapy for Capnocytophaga canimorsus infection. The figure was created by the authors using Microsoft PowerPoint (Microsoft Corp., Redmond, WA, USA).

A final diagnosis of septic shock and severe ARDS secondary to *C. canimorsus* bacteremia was established. The patient received cefotaxime soon after admission, and the culture results were positive for *C. canimorsus* five days later. Due to logistical delays associated with the transfer of samples and results from tertiary centers to regional hospitals, there was a 24-hour delay in adjusting antimicrobial coverage. On hospital day 6, the treatment was changed to broad-spectrum antibiotics (piperacillin-tazobactam and vancomycin). Due to the patient's deteriorating condition that progressed to septic shock and worsened respiratory failure, escalation to meropenem and metronidazole was necessary at day 12. An Infectious Diseases consultation was requested; the team recommended maintaining dual antimicrobial coverage in the absence of antibiotic susceptibility testing. The chronology of microbiological findings and antimicrobial escalation is summarized in Figure 3.

**Figure 4:**
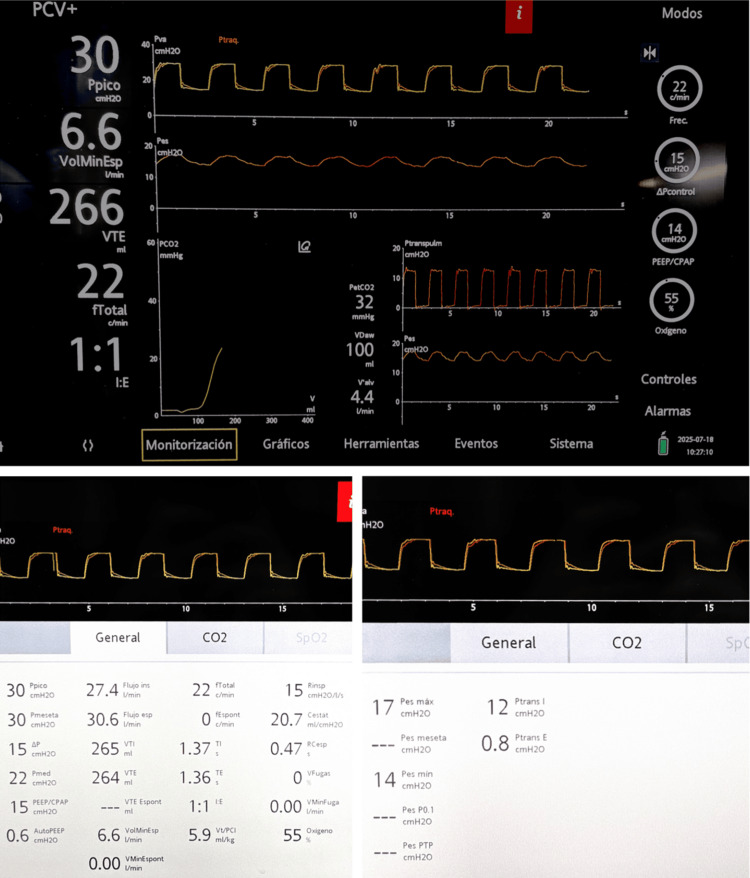
Respiratory mechanics monitoring using esophageal pressure and volumetric capnography after prone positioning.

Concurrently, the patient required mechanical ventilation with lung-protective ventilation, recruitment maneuvers using an esophageal pressure balloon and volumetric capnography, active humidification, prone positioning, neuromuscular blockade, and corticosteroids as demonstrated in the respiratory mechanics monitoring shown in Figure 4.

Due to refractory hypoxemia, VV-ECMO was initiated on day 16 of hospitalization, using femoral venous drainage and right internal jugular venous return cannulation. Initial ECMO settings included a blood flow of 3.5 L/min at 1,740 rpm, a sweep gas flow of 3 L/min, and a fraction of inspired oxygen (FiO₂) of 100%. Ultra-protective lung ventilation strategies were concurrently implemented, with a tidal volume of 4 mL/kg, respiratory rate of 10 breaths per minute, inspiratory transpulmonary pressure of 11 cmH₂O, expiratory transpulmonary pressure of 2 cmH₂O, ΔPTP of 9 cmH₂O, and a mechanical power of 3.8 J/min.

**Figure 5:**
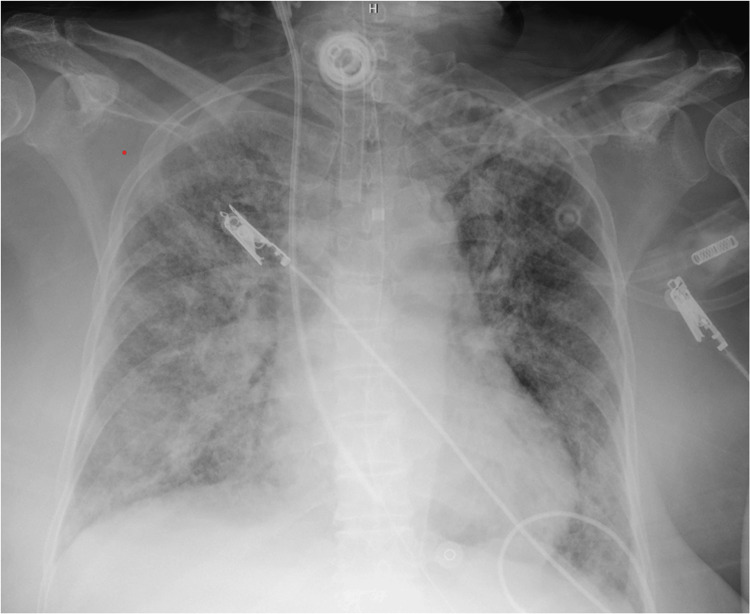
Portable anteroposterior chest radiograph obtained after veno-venous extracorporeal membrane oxygenation (VV-ECMO) decannulation demonstrating persistent diffuse bilateral pulmonary opacities.

After six days of VV-ECMO, her pulmonary compliance improved, and dead space decreased. This allowed weaning from the VV-ECMO, which was successfully discontinued after 11 days. Eighteen days after initiation of mechanical ventilation, we performed a percutaneous tracheostomy and gradually weaned the patient from mechanical ventilation. The patient experienced clinical recovery and was discharged to the referral hospital (Figure 5). During the ECMO run, the patient did not encounter any ECMO-related complications, including cannulation-related issues, circuit or console failure, neurologic events, bleeding, or thrombosis manifestations.

The patient completed 14 days of antimicrobial therapy with meropenem and metronidazole. However, owing to a catheter-related bloodstream infection due to *Klebsiella oxytoca*, meropenem was continued for an additional 14 days, through August 14.

## Discussion

The Special Bacteriology Section of the Centers for Disease Control and Prevention (CDC) in Atlanta, GA (USA), first described *C. canimorsus*. The first blood culture isolate of this species (originally called CDC group DF-2) was from a patient with bacteremia in California in 1961 [3].

In the United Kingdom, approximately 6,000 people are hospitalized annually due to dog bites. Rabies, *Pasteurella*, and *Bartonella* are among the microorganisms most associated with dog bites. Human infections by *C. canimorsus* are uncommon, with an estimated incidence of 0.67 per million inhabitants [4].

*C. canimorsus* is a commensal bacterium present in the oral flora of dogs and cats. It has also been isolated in humans following bites, scratches, licking, or even close exposure to dogs or cats. This species is a facultative, anaerobic, Gram-negative, bacillus (1-4 μm in length) that has a fusiform or filamentous shape and gliding motility. It is in the Flavobacteriaceae family and is closely related to *Fusobacterium* and *Bacteroides*. Its growth is slow on blood agar, and some studies indicated that incubation for five to 14 days may be required [1].

The prevalence of this microorganism in the oral cavity of dogs ranges from 3% to 74%, and it is more common in dogs that are older than six months, male, neutered, and small breeds. This species was also documented in the oral cavity of cats, sheep, cattle, horses, guinea pigs, and rabbits. Approximately 60% of patients with *C. canimorsus* infections reported a history of dog bite, and 24% reported another type of dog contact, such as scratches or licking. Infection following exposure to a cat is rarer (3%) [2].

This pathogen has multiple virulence factors. Unlike other *Capnocytophaga* species, it is catalase-positive, so it can degrade hydrogen peroxide within phagocytic vacuoles and survive within phagocytes. It is also resistant to the complement system, its sialidase activity enables it to obtain nutrients from surface glycoproteins of host cells, and its gliding motility allows it to traverse tissues and enter the bloodstream. It only induces a limited inflammatory response because its lipopolysaccharide (LPS) does not interact with Toll-like receptor 4, the usual receptor for bacterial LPS [1,5].

Although *C. canimorsus* is considered less virulent in healthy individuals, it can cause severe disease in immunocompromised subjects, such as patients with asplenia, a history of chronic alcohol consumption, cirrhosis, hemochromatosis, or beta-thalassemia major, and in those who smoke tobacco or are taking immunosuppressive therapy [2,6]. Patients infected with *C. canimorsus* can experience a wide spectrum of symptoms. Thus, they may present with a systemic disease, such as bacteremia, sepsis, or septic shock, or with a localized infection, such as meningitis, endocarditis, necrotizing soft tissue infection, aortitis, septic arthritis, or pneumonia [1,2]. The most common initial presentation is sepsis, and these patients typically present with fever, myalgia, diarrhea, vomiting, headache, confusion, and dyspnea [6,7]. Meningitis and soft tissue infection are the next most common presentations [2,8].

The clinical manifestations are generally more severe in immunosuppressed patients [9]. Nevertheless, a systematic review of 128 immunocompetent patients demonstrated that severe *C. canimorsus* infection may also occur in this population [1]. Although *C. canimorsus* infection is considered a zoonotic disease, epidemiological links to animals are not always documented. Butler examined the records of 484 patients and found that only 60% had a history of dog bite and only 27% had some other type of animal exposure [2]. Our patient had a history of a dog bite, and a remote history of surgical asplenia was documented soon after hospitalization. Notably, she progressed to ARDS, a clinical phenotype not commonly associated with *C. canimorsus* infection.

The diagnosis of a fulminant *C. canimorsus* infection in a patient with MOF requires the integration of epidemiological, clinical, and microbiological findings, and is often complicated by the fastidious nature of this pathogen. Blood culture is the reference method for the identification of sepsis, but the long time to culture positivity (57-96 hours in most studies) delays microbiological confirmation [6]. In addition, enriched media (chocolate agar or blood agar) and incubation with 5% CO_2_ are required [2,6]. Gram staining can identify fusiform bacilli and intracellular structures, although recovery from wound cultures is rare [6]. Definitive identification can also be achieved by molecular techniques, such as matrix-assisted laser desorption/ionization time-of-flight mass spectrometry (MALDI-TOF MS) [1,6]. PCR targeting of rRNA is particularly useful in sterile samples, such as cerebrospinal fluid [2,6,10].

Our case report emphasizes that *C. canimorsus* can cause a severe infectious process. Patients with these infections have a mortality rate of 10% to 30%, and 35% to 70% of them are admitted to an intensive care unit [1,10,11]. Therefore, early suspicion of infection and timely initiation of antibiotic treatment are essential to improve patient outcomes. *C. canimorsus* is susceptible to penicillins, clindamycin, linezolid, tetracyclines, cephalosporins, and carbapenems [2,12]. However, there are penicillin-resistant strains, in which case beta-lactamase inhibitors with penicillin derivatives are recommended [1,2]. Genomic analyses of other *Capnocytophaga* species have documented the presence of group D beta-lactamases and resistance to third-generation cephalosporins [13]. This is a potential reason for the inadequate response to cephalosporin in some patients [14,15].

Therefore, when a patient has a severe infectious process, an appropriate empirical strategy may consist of meropenem followed by de-escalation based on susceptibility testing. The recommended duration of antibiotic therapy is 14 to 21 days [6,14]. Finally, antibiotic therapy must be accompanied by appropriate resuscitation measures and organ support.

## Conclusions

Although infections by *C. canimorsus* are rare and this pathogen is commonly regarded as having low virulence, some patients experience a severe and rapidly progressive disease, as in our patient. Our patient eventually developed MOF with ARDS, an atypical clinical manifestation of *C. canimorsus* infection that was only rarely reported in the literature.

The favorable outcome of our patient underscores the value of early diagnosis, recognition of rapid clinical deterioration, and the prompt initiation of appropriate antimicrobial therapy and advanced supportive strategies, such as VV-ECMO, all of which were essential for achieving patient recovery. The unusual course and severity of disease in our patient highlight the need to raise awareness about the potential impact of this zoonosis and the importance of actively asking patients about bites, scratches, or other exposures to dogs and cats. This case provides evidence that patients with infection by *C. canimorsus* can present with severe and life-threatening disease, and reinforces the importance of using a comprehensive and multidisciplinary approach with close clinical surveillance to improve the outcomes of these patients.
